# Indexing dialysis dose for gender, body size and physical activity: Impact on survival

**DOI:** 10.1371/journal.pone.0203075

**Published:** 2018-09-07

**Authors:** Sivakumar Sridharan, Enric Vilar, Andrew Davenport, Neil Ashman, Michael Almond, Anindya Banerjee, Justin Roberts, Ken Farrington

**Affiliations:** 1 Renal Unit, Lister Hospital, Stevenage, United Kingdom; 2 University of Hertfordshire, Hatfield, United Kingdom; 3 Department of Nephrology, University College London, Royal Free Hospital, London, United Kingdom; 4 Royal London Hospital, London, United Kingdom; 5 Southend University Hospital, Westcliff-on-Sea, United Kingdom; 6 Arrowe Park Hospital, Wirral, United Kingdom; 7 Anglia Ruskin University, Cambridge, United Kingdom; Universidade Estadual Paulista Julio de Mesquita Filho, BRAZIL

## Abstract

Current practice basing dialysis dose on urea distribution volume (V) has been questioned. We explored the impact on survival of scaling dialysis dose (Kt) to parameters reflective of metabolic activity. In a multicentre prospective cohort study of 1500 patients on thrice-weekly haemodialysis, body surface area (BSA) and resting energy expenditure (REE) were estimated using validated equations and physical activity by the Recent Physical Activity Questionnaire. Total energy expenditure (TEE) was estimated from REE and physical activity data. Kt was calculated from delivered (single-pool Kt/V)*Watson V. Kt/BSA, Kt/REE and Kt/TEE were then calculated at baseline and 6 monthly during follow-up for 2 years. In adjusted Cox models Kt/TEE, Kt/BSA, Kt/REE, in that order, had lower hazard ratios for death than single-pool Kt/V. On the basis of adjusted survival differences, putative minimum target doses were estimated for Kt/BSA as 27119 ml/m^2^ and Kt/TEE as 25.79 ml/kcal. We identified spKt/V values equivalent to these estimated targets, ranging from 1.4 to 1.8 in patient groups based on gender, body size and physical activity. For sedentary patients, the minimum target dose was 1.4 for large males, 1.5 for small males and 1.7 for women. For active patients the target was 1.8 irrespective of gender and body-weight. Patients achieving these individualised minimum targets had greater adjusted two-year survival compared to those achieving conventional minimum targets. Metabolic activity related parameters, such as Kt/TEE and Kt/BSA, may have a clinically important role in scaling haemodialysis dose. Using such parameters or their spKt/V equivalents to adjust minimum target doses based on gender, body size and habitual physical activity may have a positive impact on survival.

## Introduction

The current paradigm of measuring dialysis adequacy centres on small solute clearance, using urea as the marker solute. Dialysis dose (Kt/V) is construed as urea clearance (K) during a dialysis session of duration (t), scaled to the urea distribution volume which is equivalent to total body water volume (V). There have been suggestions that this approach may lead to under-dialysis in women and small men [1–3]. This has stimulated a search for alternative scaling parameters [4]. Parameters such as body surface area (BSA), resting energy expenditure (REE) and total energy expenditure (TEE) have been proposed [5]. However, prospective studies comparing the impact of these parameters on survival are not available.

Scaling dialysis dose (Kt) based on BSA may provide more dialysis to women and small patients, and has been suggested as a potential improvement on Kt/V [6–8]. Indeed, retrospective analysis of cohorts of haemodialysis patients have reported improved survival with greater delivered dialysis dose when dialysis dosing was rescaled to BSA rather than V [9].

REE and TEE reflect metabolic activity closely and hence, could also be considered as alternative scaling parameters [10,11]. REE and TEE are related to body composition, in particular lean body mass and skeletal muscle mass [12,13]. Increased energy expenditure is associated with higher levels of urea generation rate in haemodialysis (HD) patients [14]. In normal individuals there is a strong correlation between energy expenditure and protein turnover. Indeed in healthy older patients the proportion of energy expenditure associated with protein turnover is around 45% [15]. TEE, which encompasses physical activity-related energy expenditure, may capture the sum total of metabolic activity better and thereby total metabolic waste production against which the dialysis dose could be scaled.

Our aim in this study was to compare dialysis dose scaled using V, BSA, REE and TEE in relation to survival outcomes. We also aimed to estimate putative minimum target doses in terms of these alternative parameters in relation to gender, body size and physical activity levels.

## Subjects and methods

### Ethical review

The study was approved by the North Wales Regional Ethics Committee. All subjects gave informed written consent.

### Subjects

An analysis of baseline data has been described previously which included characterization of the study cohort [5]. The study took place in five UK centres. Subjects were recruited from March 2012 through to December 2012 and followed up till the completion of the study period or until a censored event occurred as mentioned below.

Prevalent maintenance haemodialysis patients older than 18 years who had been receiving dialysis for more than 3 months were recruited. Exclusion criteria included patients dialyzing on schedules other than thrice-weekly, those with amputated limbs and those with no capacity to consent. The patient information sheet, consent form, and the questionnaires were translated into Bengali and Urdu to facilitate inclusion of non-English-speaking patients.

### Baseline data collection

The following baseline data were obtained for each patient.

1. Demographic data including age, sex, ethnicity and dialysis vintage was collected from medical records
2. Height and pre- and post-dialysis weight were collected by direct measurement
3. Pre- and post-dialysis biochemistry and haematology results were obtained from routinely collected data.
4. Comorbidity data was collected by using a self-report questionnaire described previously [16]. This scale is based on the presence and severity (grades 1–3) of seven conditions: arthritis, cancer, diabetes, heart disease, lung disease, liver disease, and stroke (maximum score 21). Patients with a score ≥ 3 were designated as having high comorbidity. Scores generated from this questionnaire have similar predictive power for short term mortality as the Charlson Comorbidity Index [16].
5. Physical activity data was obtained using the Recent Physical Activity Questionnaire (RPAQ) which has been validated in the general population [17] and in CKD patients [18]. Each reported activity was assigned a metabolic equivalent task (MET) value with reference to the Compendium of Physical Activities [19,20]. The mean daily MET value was obtained by summing the individual values for each activity (Total Daily MET) and dividing by 24 hours. To examine the effect of activity level on survival, patients were categorised into 3 groups based on daily MET value. Those with MET value of ≤1.2 kcal/kg/h were categorised as sedentary, those with 1.2–1.5 kcal/kg/h as Light Active, and those with >1.5 kcal/kg/h as Active.

### Follow-up

Patients were followed-up for 24 months or until death, transplantation, change of dialysis modality, recovery of renal function or transfer to another centre. Date of death or other censoring event was recorded. Follow-up data was collected at 3, 6, 12, 18 and 24 months from recruitment. Pre- and post-dialysis weight and routine pre- and post-dialysis biochemistry results were also collected during follow-up.

### Scaling parameters

The following values were derived at each time point.

V—using the Watson equations [21]

BSA–using the Haycock formula [22]

REE–using an equation derived and validated in haemodialysis patients [23]

TEE—estimated as previously described [18] from the following equation:

TEE(kcal/day)=REExMeandailyMET

### Estimation of adequacy parameters

Dialysis was prescribed to achieve a minimum target single pool Kt/V (spKt/V) of 1.2 in four units and an equilibrated Kt/V of 1.1 in the fifth centre. Delivered spKt/V at each time point during follow-up was calculated from pre- and post-dialysis parameters using the Daugirdas formula [24].

Values of delivered Kt corresponding to spKt/V at each time point during follow-up were calculated using the following formula.

$$Kt=Delivered\frac{spKt}{V}*V$$

Values of delivered Kt/BSA, Kt/REE and Kt/TEE corresponding to delivered spKt/V were then derived by dividing Kt at each time point by the respective alternative scaling parameters at that time point.

### Standardisation

In order to allow direct comparison between various dialysis adequacy parameters in Cox survival models, standardised variables of each of the adequacy parameters were derived by the following method.

$$Standardised\frac{spKt}{V}=\frac{\left(\frac{spKt}{V}-\mu \right)}{\sigma }$$

where spKt/V is the parameter value for each patient, μ is the population mean of Kt/V and σ is the standard deviation of Kt/V in the study population.

Standardised Kt/BSA, Kt/REE and Kt/TEE were calculated similarly using the respective parameter and population means and standard deviations.

### Statistics

Statistical analysis was carried out using SPSS ^®^ version 24 (SPSS Software, IBM Corporation, New York, USA) and Prism 7.0 (GraphPad, San Diego, USA). Normally distributed data are presented as mean ± SD, and non-normally distributed as median [interquartile range]. The significance of differences between means was determined by Student’s t-test and between medians by the Mann-Whitney U test. A p-value of <0.05 was assumed to indicate statistical significance. Independent predictors of survival were assessed using Cox regression models. Since delivered dialysis dose changed with time, all of the adequacy parameters were computed as time-dependent covariates in the models. Cut-off points were determined for the values of Kt/BSA and Kt/TEE below which mortality rates were significantly higher through separate Cox models of survival based on categorized variables of these parameters–tertiles, quartiles, quintiles and sextiles. Each model was adjusted for age, sex, ethnicity, comorbidity, dialysis vintage, body mass index (BMI) and physical activity level. Linear regression models were then constructed to estimate values of spKt/V equivalent to these threshold values in groups of patients defined by sex, body-weight, and physical activity level. Adjusted survival was then determined for patients according to their achievement of these putative targets and conventional spKt/V targets.

## Results

A total of 1500 patients were recruited. Mean baseline demographic, anthropometric and scaling parameters are shown in Table 1 [5].

**Table 1:** Demographic, anthropometric and energy metabolism characteristics of 1500 study patients.

	All Patients (n = 1500)	Males (n = 910)	Females (n = 590)	p-value
**Age (years)**	62.9 ± 15.5	63.8 ± 15.6	61.6 ± 15.1	0.007
**Weight (kg)**	75.2 ± 18.3	78.4 ± 17.3	70.4 ± 18.6	<0.001
**Height (cm)**	165.9 ± 10.0	170.6 ± 8.2	158.7 ± 8.2	<0.001
**Ethnicity (% Asian: Black: White)**	27.9: 26.7: 45.5	27.4: 24.0: 48.7	28.6: 30.8: 40.5	0.003
**Body Mass Index (kg/m**^**2**^**)**	27.3 ± 6.0	26.9 ± 5.3	27.9 ± 7	0.002
**High Comorbidity (%)**	29.5	28.7	30.7	NS
Dialysis vintage (years)*	3.2 (4.6)	3.0 (4.3)	3.6 (5.0)	0.01
Mean Daily MET*	1.17 (0.09)	1.17 (1.54)	1.16 (0.08)	0.001
**Watson Volume (L)**	37.4 ± 7.3	40.8 ± 6.1	32.1 ± 5.0	<0.001
**BSA (m**^**2**^**)**	1.86 ± 0.26	1.93 ± 0.24	1.76 ± 0.26	<0.001
**REE (kcal/day)**	1541 ± 250	1616 ± 229	1426 ± 236	<0.001
**TEE (kcal/day)**	1837 ± 388	1943 ± 391	1673 ± 322	<0.001
**Kt/V**	1.57 ± 0.27	1.51 ± 0.25	1.68 ± 0.28	<0.001
**Kt/BSA (ml/m**^**2**^**)**	27,217 ± 4192	27,740 ± 4042	26,412 ± 4294	<0.001
**Kt/REE (ml/kcal)**	32.99 ± 5.16	33.11 ± 4.73	32.81 ± 5.76	0.278
**Kt/TEE (ml/kcal)**	28.02 ± 5.05	27.92 ± 4.78	28.18 ± 5.43	0.342

REE: resting energy expenditure, TEE: total energy expenditure. Mean daily MET: mean daily metabolic equivalent of task (kcal/kg/h). Values expressed as mean ± SD and as median (interquartile range) for variables that were not normally distributed (marked as *). Proportions of categorical variables are expressed as percentages.

### Dialysis dose in relation to gender, body size and physical activity

All body size parameters as well as REE and TEE were lower in women than men. Mean delivered spKt/V was higher in women, whilst Kt/BSA was higher in men. There were no gender differences for Kt/REE or Kt/TEE. Mean delivered dose was higher in small (< median weight) than in large men, irrespective of scaling parameter. There were significant differences in delivered dialysis dose between the sedentary, light active and active groups, the degree depending on the scaling parameter used (Table 2). The most active individuals (daily MET >1.5 kcal/kg/h) received similar doses (± 5%) to those less active, when using spKt/V, Kt/BSA and Kt/REE, but over 40% less dialysis when adjusted to TEE. Physical activity was generally low, 73% being sedentary (daily MET < 1.2 kcal/kg/h), with only 4% in the active category. Pre-dialysis serum urea was also related to activity; 18.9 ± 5.7 mmol/L for sedentary, 19.9 ± 5.6 mmol/L lightly active and 21.9 ± 5.8 mmol/L for active patients (p < 0.001 by one-way ANOVA).

**Table 2:** Differences in mean delivered dialysis dose expressed using various scaling parameters between patients with different levels of physical activity categories.

Parameter	Sedentary (n = 1100)	Light Active (n = 346)	Active (n = 54)	p-value
Kt/V	1.58 ± 0.27	1.57 ± 0.28	1.50 ± 0.27	0.57*; 0.09^†^; 0.04^§^
Kt/BSA (ml/m^2^)	27,031 ± 4204	27,600 ± 4025	28,569 ± 4651	0.03*; 0.15^†^; 0.02^§^
Kt/REE (ml/kcal)	33.06 ± 5.28	32.88 ± 4.82	32.34 ± 4.90	0.58*; 0.44^†^; 0.32^§^
Kt/TEE (ml/kcal)	29.18 ± 4.76	25.61 ± 4.02	19.84 ± 3.54	< 0.001*^†^^§^

V: Total Body Water Volume, BSA: Body Surface Area, REE: resting energy expenditure, TEE: total energy expenditure. T-test comparison between *sedentary and light active groups

^†^light active and active groups

^§^sedentary and active groups

### Dialysis dose and survival

There were 316 deaths during the 24 month follow-up. Two hundred and thirty five patients were censored for various reasons including 179 for renal transplantation. Unadjusted 2-year survival was better for women than men (81% vs 74%; p = 0.001), non-Whites vs Whites (84% vs. 68%; p < 0.001) and for those with low vs high comorbidity (81% vs. 67%; p < 0.001).

The four scaling parameters were standardised as described in the Methods section and entered as time-dependent covariates into separate Cox proportional hazard models. Table 3 shows models 1 to 4. Model 1 included standardised spKt/V. Model 2 used the same covariates with Kt/BSA substituted for spKt/V. Model 3 used the same covariates with Kt/REE substituted for spKt/V. Model 4 used the same covariates with Kt/TEE substituted for spKt/V. Each of the alternative adequacy parameters contributed significantly to their respective model. In these models standardised Kt/TEE and Kt/BSA had the lowest Hazard ratios. These parameters were used in subsequent analyses.

**Table 3:** Cox models of survival using Standardised spKt/V as time-dependent covariate (Model 1), standardised Kt/BSA as time-dependent covariate (Model 2), standardised Kt/REE as time-dependent covariate (Model 3) and standardised Kt/TEE as time-dependent covariate (Model 4).

Variables	p-value	Hazard Ratio	95% confidence interval for Hazard Ratio	
			Lower	Upper
**MODEL1**				
Age (years)	< 0.001	1.028	1.018	1.038
Female Gender	0.158	0.836	0.652	1.072
Asian Ethnicity	0.056	0.767	0.585	1.007
Black Ethnicity	<0.001	0.402	0.284	0.569
Comorbidity Score	< 0.001	1.143	1.089	1.200
Dialysis Vintage (years)	0.035	1.028	1.002	1.054
BMI (kg/m^2^)	< 0.001	0.955	0.934	0.976
Mean Daily MET	0.003	0.103	0.024	0.450
Standardised spKt/V	0.001	0.813	0.720	0.918
**MODEL 2—substituting Kt/BSA for spKt/V in MODEL 1**				
Kt/BSA	<0.001	0.714	0.636	0.802
**MODEL 3—substituting Kt/REE for spKt/V in MODEL 1**				
Kt/REE	<0.001	0.718	0.641	0.805
**MODEL 4—substituting Kt/TEE for spKt/V in MODEL 1**				
Kt/TEE	<0.001	0.709	0.628	0.801

BMI = Body Mass Index, MET = Metabolic Equivalent of Task. Units for variables are mentioned above where present. Comorbidity score and Mean daily MET do not have units. All standardised dialysis dose variables have unit of one standard deviation.

### Estimating minimum target dialysis dose in terms of Kt/BSA and Kt/TEE

**Fig 1:**
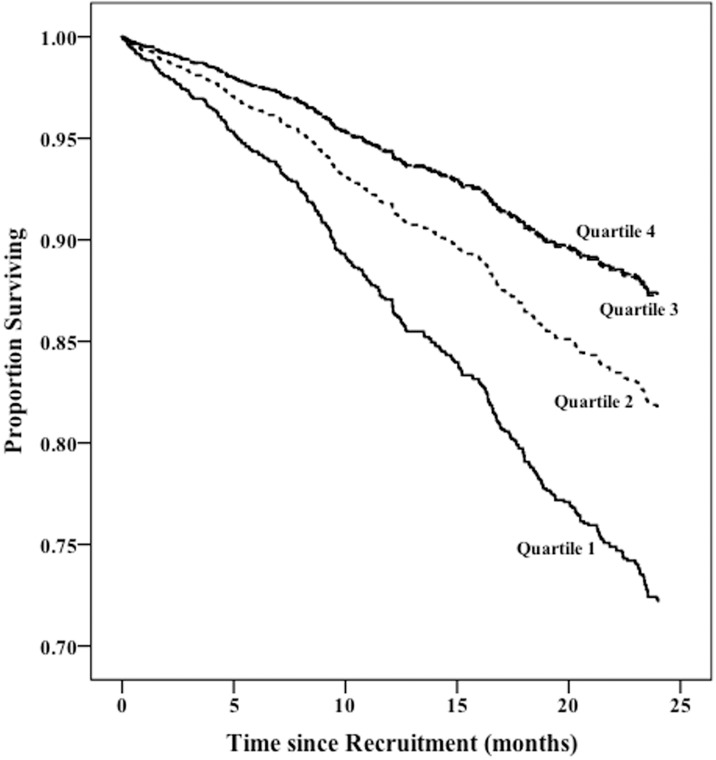
Survival Plot based on Kt/BSA quartiles adjusted for age, sex, ethnicity, comorbidity, dialysis vintage, BMI and physical activity level. Lines for Quartiles 3 and 4 overlap and hence they are not delineated in the graph.

**Fig 2:**
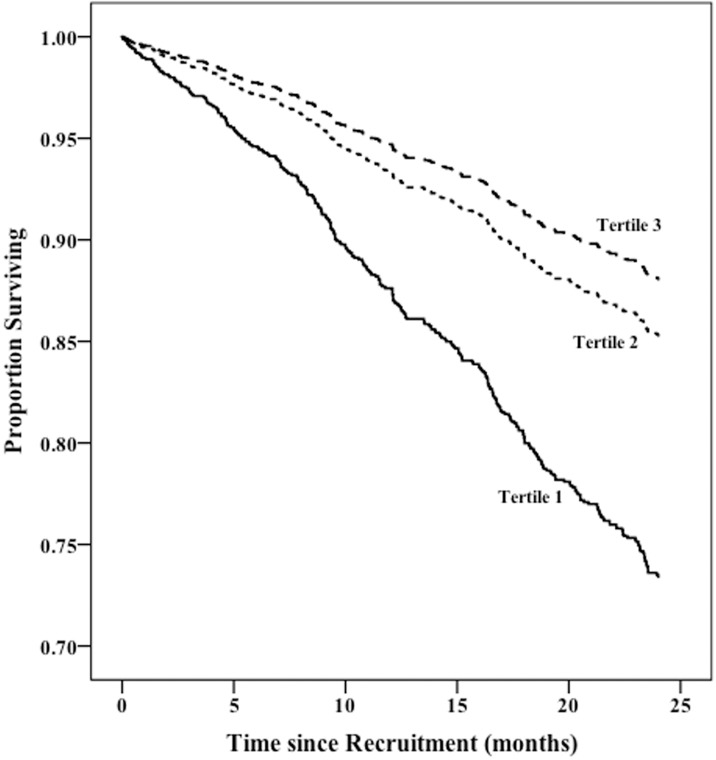
Survival plot based on Kt/TEE tertiles adjusted for age, sex, ethnicity, comorbidity, dialysis vintage, BMI and physical activity level.

As described in methods, we built separate Cox models of survival based on categorized variables of Kt/BSA and Kt/TEE–tertiles, quartiles, quintiles and sextiles. For Kt/BSA the best model showed that patients in the bottom two quartiles i.e. those with Kt/BSA less than the median level of 27119 ml/m^2^ had significantly reduced adjusted survival compared to those in the top two quartiles (Fig 1). A larger proportion of women were present in the bottom 2 quartiles compared to the proportion of men in the same groups (59.2% vs. 44.1%; p < 0.001) Fig 2 shows the survival differences based on Kt/TEE. The best model showed that patients in the lowest tertile i.e. those with Kt/TEE less than 25.79 ml/kcal had significantly worse adjusted survival. The lowest tertile contained a larger proportion of lightly active and active patients compared to that of the sedentary group (60.3% vs. 23.5%; p < 0.001).

### Estimating Kt/V equivalent to minimum target Kt/BSA and Kt/TEE in relation to gender, body size and physical activity levels

We then plotted spKt/V versus Kt/BSA according to gender specific weight to estimate equivalent doses in 4 patient groups (males and females, above and below median weight) (Table 4). Linear regression equations were constructed, intercepts, slopes and R^2^ values of which are shown in Table 4. The equations were solved for a Kt/BSA value of 27119, as patients with levels below this had significantly reduced adjusted survival. We then used this cut off value to define the equivalent levels of spKt/V in these four patient groups. In women, there was no effect of weight on dialysis dose and the equivalent spKt/V dose was 1.7 for both weight groups, whereas the equivalent spKt/V dose was 1.4 in larger men and 1.5 in smaller men.

**Table 4:** Equivalent minimum target spKt/V levels to Kt/BSA of 27119 in 4 patient groups defined by gender and weight (less than or greater than median).

Patient Group	Frequency	Intercept	Slope	R^2^	Equivalent spKt/V
**Females**					
Small	295 (19.7%)	0.49	0.0000462	0.572	1.7
Large	295 (19.7%)	0.19	0.0000554	0.686	1.7
**Males**					
Small	455 (30.3%)	0.15	0.0000506	0.717	1.5
Large	455 (30.3%)	0.07	0.0000501	0.713	1.4

The intercept, slope and R^2^ values relate to linear regression equations relating spKt/V and Kt/BSA. These were solved for Kt/BSA = 27119 to yield suggested minimum target spKt/V level (rounded to one decimal place).

We then performed a similar analysis of spKt/V versus Kt/TEE (Table 5) in 3 patient groups based on activity level as described above. The equations were solved for Kt/TEE of 25.79 as this was the threshold associated with increased mortality. The equivalent minimum spKt/V was 1.4, 1.6 and 1.8 for sedentary, light active and active groups respectively.

**Table 5:** Equivalent minimum target spKt/V levels to Kt/TEE of 25.79 in 3 patient groups based on activity level.

Patient Group	Frequency	Intercept	Slope	R^2^	Equivalent spKt/V
Sedentary	1100 (73.3%)	0.39	0.04	0.511	1.4
Light Active	346 (23.1%)	0.29	0.05	0.510	1.6
Active	54 (3.6%)	0.52	0.05	0.404	1.8

The intercept, slope and R^2^ values relate to linear regression equations relating spKt/V and Kt/TEE. These were solved for Kt/TEE = 25.79 to yield suggested minimum target spKt/V level (rounded to one decimal place).

These two targets (Tables 4 and 5) were then combined to estimate an individualized minimum target dose based on gender, body size and physical activity level. For each category, the highest corresponding spKt/V value was used from Tables 4 and 5. Using this method, then sedentary patients would not require any additional adjustment of spKt/V for activity level (Table 6). Large men, who were lightly active, would need an upward adjustment of 0.2 and those who were active would need an upward adjustment of 0.4. For small men, this would be 0.1 for lightly active patients and 0.3 for active patients. Female patients would need an upward spKt/V adjustment of 0.1, only if they were in the active group.

**Table 6:** Recommended minimum spKt/V target based on gender, body size and activity level adjustment.

	Sedentary	Light Active	Active
**Large males**	1.4 (n = 309)	1.6 (n = 120)	1.8 (n = 26)
**Small males**	1.5 (n = 319)	1.6 (n = 116)	1.8 (n = 20)
**Females**	1.7 (n = 472)	1.7 (n = 110)	1.8 (n = 8)

### Adjusted survival in relation to gender, body size and activity specific targets

**Fig 3:**
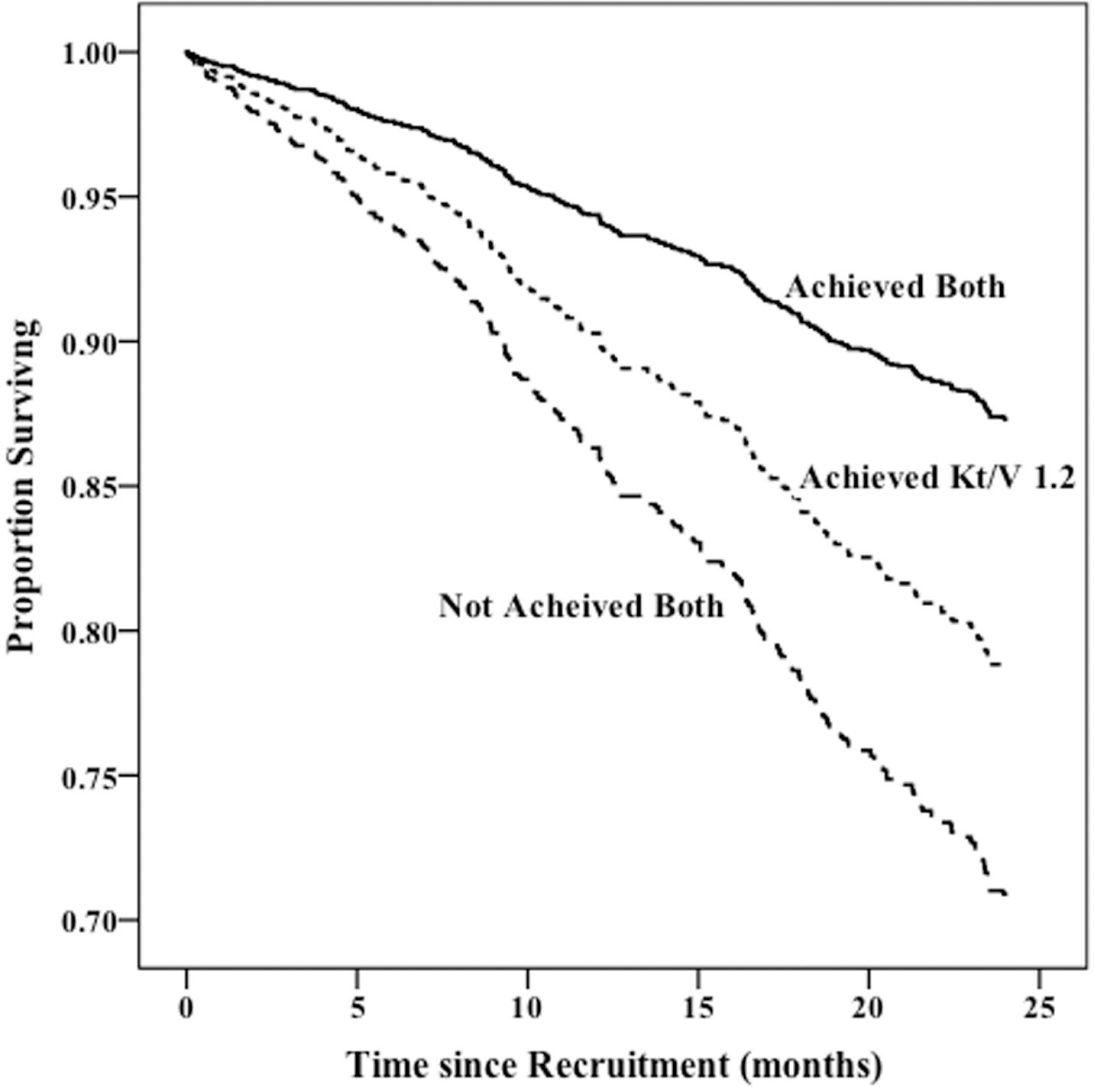
Adjusted survival in patients according to achievement of recommended spKt/V adequacy targets based on gender, body size and physical activity and by conventional criteria (spKt/V >1.2). The lines represent whether the patient has met both recommended dose criteria and conventional, just the conventional or neither.

We then compared survival, adjusted for the same parameters as in our previous models, in patients according to their achievement of minimum spKt/V target levels defined by conventional criteria (spKt/V > 1.2) and by the spKt/V target equivalent to the recommended value shown in Table 6. Adjusted 2 year survival was 87% in the 49% of patients who exceeded both the conventional spKt/V and the adjusted targets based on the Kt/TEE and Kt/BSA equivalent, 79% in the 43% who exceeded the conventional spKt/V target but not the recommended equivalent target, and 71% in the 8% who failed to achieve both targets (Fig 3).

## Discussion

Our findings suggest that though spKt/V delivery is higher in women than men, normalizing clearances by parameters more reflective of metabolic activity–BSA, REE, TEE–abolished or reversed this difference. The small proportion of patients who were active (daily MET >1.5 kcal/kg/h) in our cohort received similar doses (± 5%) to those who were less active according to spKt/V, Kt/BSA and Kt/REE, but received a much lower dose, more than 40% lower—when scaled to Kt/TEE. Kt/BSA, Kt/REE and Kt/TEE may be better predictors of adjusted survival than Kt/V, the hazard ratio for mortality was lowest when scaling dialysis dose by TEE, followed by BSA and REE. Scaling by REE provided no advantage over BSA.

We estimated putative minimum target doses using these alternative scaling parameters on the basis of differences in adjusted survival. For Kt/BSA estimated minimum target was 27119 ml/m^2^ and for Kt/TEE 25.79 ml/kcal. Adjusted spKt/V levels equivalent to these targets ranged from 1.4 to 1.8 according to gender, body size and physical activity. This suggests that current minimum spKt/V target of 1.2 may result in under-dialysis in all our patient groups. Light active and active patients would need a higher spKt/V target depending on gender and body size. Achievement of these estimated targets may confer a survival advantage over and above that conferred by achievement of the current conventional minimum targets.

In current practice, total body water volume (V) is the denominator by which dialysis dose is scaled. Due to differences in body composition, V is significantly lower in women than men leading to inflated Kt/V values for similar dialysis doses based on parameters more reflective of metabolic activity. V is linearly related to body-weight whilst basal metabolic rate (BMR) and related parameters e.g. BSA and REE–are related to body-weight (W) by power functions of the form CW^b^, where C is a constant and b < 1 [25–27]. This reflects the non-linear relationship of visceral organ metabolism, the major contributor to BMR, to body-weight and contributes to the relatively higher concentrations of metabolic waste/kg in smaller individuals [28]. These smaller individuals tend to require higher minimum target Kt/V levels. Use of BSA (or REE) as the scaling factor would abrogate the need for such adjustments. Neither of these parameters however, takes into account energy expenditure related to physical activity. Use of TEE, which encompasses both BMR and physical activity-related energy expenditure, reflects total metabolic waste production. Taking physical activity into account in dialysis dosing is not part of current recommendations. The impact of failing to account for physical activity may have been minimized by the habitually low levels of physical activity undertaken by the dialysis population–though the small proportion of relatively active individuals may have been compromised. A holistic approach, providing adequate dialysis dosing across all patient groups would take account of gender, body size and physical activity.

Although, the current guidelines suggest that the minimum spKt/V target of 1.2 may have to be higher in women than men, they fall short of stating gender-specific minimum targets for dialysis dosing [29]. Previous reports have shown similar survival on haemodialysis of women and men [30], in contrast to the general population where women have a survival advantage. We found unadjusted survival to be higher in women. This may be due to more women in our study receiving delivered dialysis doses above the Kt/BSA and Kt/TEE thresholds.

This is the first prospective comparison study of these metabolic parameters for dialysis dosing in relation to survival in the haemodialysis population. The study recruited a large cohort of patients, representative of the dialysis population, with an adequate representation from different ethnic groups. There were however a number of limitations. This was an observational cohort study of prevalent patients with relatively short follow-up. Hence it may not be safe to extrapolate findings to the longer term. Estimations of scaling parameters were carried using anthropometric readings obtained at baseline. However, these parameters were measured and not derived from medical records. Watson volume was estimated using anthropometric measures since most of the participating centres did not use urea kinetic modelling or bioimpedance measured V to prescribe dialysis. Hence, modelled or bioimpedance estimated V was not used in the prescription for the majority of study subjects in keeping with routine clinical practice. As there may be differences between the estimation of body water using the Watson anthropomorphic equation and that measured by bioimpedance, this may potentially have led to an overestimation of Kt in our study. As with any questionnaire-related data acquisition, recall bias is a potential confounder in the accuracy of TEE data. In addition these data rely on estimates of daily MET levels derived from the RPAQ instrument. The results may vary with use of alternative activity questionnaires. Due to the nature of the study and because dialysis was prescribed based on Kt/V, it was not possible to statistically compare the hazard ratios shown for each of the parameters to assess whether the differences noted were indeed significant. Residual renal function was not routinely measured in most of the 5 participating centres and hence, this was not included in survival analyses. As this was an observational study, no intervention was carried out to maintain dialysis dose within a range specified as part of the study. This, along with varying physical activity levels, might be a confounding factor as some patients would have received higher than their intended dialysis dose. Finally, we should stress that these estimated adequacy targets relate only to urea clearance. Many other factors such as clearance of middle-molecules and protein-bound solutes and fluid volume status are also important in survival in haemodialysis patients. Clearly though, whatever other parameters are used, it is necessary to ensure adequate clearance of metabolic waste products.

In conclusion, metabolic activity related parameters, particularly Kt/TEE and Kt/BSA, may have an important role in scaling haemodialysis dose. Using such parameters or their adjusted spKt/V equivalents to deliver minimum target doses based on gender, body size and habitual physical activity may have a positive impact on survival. Future trials of dialysis dosing should consider metabolic activity.
